# Suicidal ideation in Chinese adolescents: prevalence, risk factors, and partial mediation by family support, a cross-sectional study

**DOI:** 10.3389/fpsyt.2024.1427560

**Published:** 2024-08-02

**Authors:** Xiyan Bao, Tianming Guo, Li Xu, Wanming Chen, Lingshu Luan, Haidong Yang, Xiaobin Zhang

**Affiliations:** ^1^ Department of Sleep Medicine, The Fourth People’s Hospital of Yancheng, Yancheng, China; ^2^ Department of Clinical Medicine, Xuzhou Medical University, Xuzhou, China; ^3^ Department of Psychiatry, The Fourth People’s Hospital of Lianyungang, The Affiliated KangDa College of Nanjing Medical University, Lianyungang, China; ^4^ Medical College of Yangzhou University, Yangzhou, China; ^5^ Institute of Mental Health, Suzhou Psychiatric Hospital, The Affiliated Guangji Hospital of Soochow University, Suzhou, China

**Keywords:** suicidal ideation, mediator, GAD-7, PSSS, adolescents

## Abstract

**Background:**

Suicidal ideation is a pressing public health concern, particularly among adolescents. The objective of this study was to examine the prevalence of and factors associated with suicidal ideation in Chinese adolescents, addressing an important gap in current research.

**Methods:**

This study employed an online survey of 3443 adolescents in Lianyungang, using a cross-sectional design. The assessment included the use of the Patient Health Questionnaire-9, the seven-item Generalized Anxiety Disorder instrument, and the Perceived Social Support Scale to evaluate suicidal ideation, anxiety symptoms, and social support in adolescents, respectively.

**Results:**

In adolescents, the prevalence of suicidal ideation was 22.1%, with a significantly higher proportion among female adolescents than among males (27.9% vs 16.9%, P < 0.001). Binary regression analysis identified (OR = 1.788, 95% CI: 1.467–2.177, P < 0.001), anxiety symptoms (OR = 10.035, 95% CI: 7.441–13.534, P < 0.001), total PHQ-9 scores of mothers (OR = 1.040, 95%CI: 1.003 – 1.078, P = 0.034), total GAD-7 scores of mothers (OR = 0.958, 95%CI: 0.919 – 0.998, P = 0.039), and moderate parental relationships (OR = 2.042, 95% CI: 1.630–2.557, P < 0.001) to be risk factors for suicidal ideation; family support was a protective factor (OR = 0.888, 95% CI: 0.859–0.918, P < 0.001). Furthermore, family support partially mediates the relationship between anxiety symptoms and suicidal ideation among adolescents (9.28%).

**Conclusions:**

This study highlights high adolescent suicidal ideation rates and recommends gender-specific interventions, anxiety management, and family support for improvement in mental health status.

## Introduction

Adolescent is a pivotal developmental phase marked by profound physical, emotional, and social transformations (1). Although many adolescents navigate these transitions smoothly, a significant subset grapple with difficulties, culminating in detrimental effects on their mental health (2). The mental health landscape among adolescents has garnered increasing global concern in recent years. One particularly concerning outcome requiring urgent scrutiny is suicidal ideation, which encompasses thoughts and contemplation of self-harm or suicide (3, 4). According to a previous large-sample survey, the lifetime prevalence of suicidal ideation among pre-adolescent children was approximately 15.2%, with close to 17.0% of suicidal thoughts turning into suicide attempts (5). National and regional statistical data on adolescent suicide mortality rates and specific causes in China are scarce, which to some extent reflects the challenges in collecting and publicizing such data. Nevertheless, the study by Liu et al. provides some valuable insights (6). They reported that the suicide mortality rate among individuals aged 15-29 in western China from 2006 to 2018 was 19.9 per 100,000 population, with no significant difference between males and females (14.0 and 13.4 per 100,000, respectively). The study also identified higher education and previous suicide attempts as strong risk factors for suicide. Such a phenomenon necessitates immediate attention and calls for thorough investigation to mitigate its pervasive impact on this vulnerable population (7).

Suicide ranks as the fourth leading cause of death globally among adolescents and young adults aged 15 to 29 (8). Studies conducted in various countries have revealed alarming rates of suicidal ideation or suicide attempts among adolescents. For example, in one study, the reported prevalence of suicidal ideation among adolescents aged 13–19 years was 24.66%, and the reported prevalence of suicide attempts was 4.37% (9). Additionally, the range of reported prevalence values for suicide attempts among adolescents aged 12–15 years varies from 1.2% to 13.8% (10). A significant proportion of self-reported suicidal ideation among teenagers occurs frequently or consistently, posing a direct risk to their mental health. A cross-sectional study demonstrated that 51.6% of students aged 13–16 reported experiencing suicidal thoughts, with 32.2% reporting active suicidal thoughts within the two weeks preceding the survey (11). Examining the prevalence of suicidal ideation is crucial for developing a comprehensive understanding of this multifaceted phenomenon. Despite the variations, it is evident that suicidal ideation represents a prevalent concern globally.

The correlates of suicidal ideation are complex, and previous studies have found various factors to be associated with it, including psychiatric disorders (such as depression and anxiety), a history of self-harm, social isolation, and adverse childhood experiences (12–14). A cluster of randomized controlled studies found that worry, loss of sleep, and anxiety were significant predictors of the risk of suicidal ideation and behavior among youths aged 12–17 years, specifically for an 18-month timeframe (15). A study conducted by Guedria-Tekari et al. revealed that smoking, non-suicidal self-injurious behavior, and low self-esteem were recognized as risk factors associated with suicidal thoughts in adolescents (16). Hesketh et al. found that suicidal ideation among Chinese adolescents was associated with female gender, poor self-reported academic performance, and rural residence (17). Only 1% of students sought professional help, with most relying on friends and parents for support. This indicates that parental or familial support plays a protective role against adolescent suicidal ideation. Additionally, recent studies among Chinese adolescents have shown that non-suicidal self-injury mediates suicidal ideation (18), and there is a significant positive correlation between harsh parenting and adolescent suicidal ideation (19). Self-esteem mediates the relationship between harsh parenting and adolescent suicidal ideation, and school social support moderates the indirect effect of harsh parenting on suicidal ideation (19). These findings further illustrate the complexity of suicidal ideation among adolescents.

In terms of adolescent anxiety symptoms and suicidal ideation, the mediating role of social support proves to be complex and pivotal (20). Social support encompasses various forms of assistance, comfort, and companionship available from individuals within an individual’s social network, such as family members, friends, or peers (21, 22). In addition, more extensive social support serves as a buffer against the development and persistence of both anxiety symptoms and suicidal ideation in adolescents (23, 24). Negligent and affectless control in the family, insecure attachment, and stressful life events are risk factors for suicide, leaving parental care and security-giving as protective factors (25). Adolescents who experience loneliness are 37.6% more likely to develop sleep disorders and have a 21% higher likelihood of experiencing suicidal ideation when compared with the general population (26).

For suicide in adolescents, understanding the prevalence and risk factors is crucial for effective prevention and intervention efforts in addressing the challenges posed by suicidal ideation. Accordingly, the purpose of this study was to investigate 1) the prevalence of suicidal ideation and associated risk factors and 2) whether social support played a mediating role in the relationship between anxiety symptoms and suicidal ideation in adolescents. Through this research, our aim was to make a meaningful contribution to the continually growing body of knowledge surrounding suicidal ideation among adolescents.

## Materials and methods

### Participants and procedures

**Figure 1:**
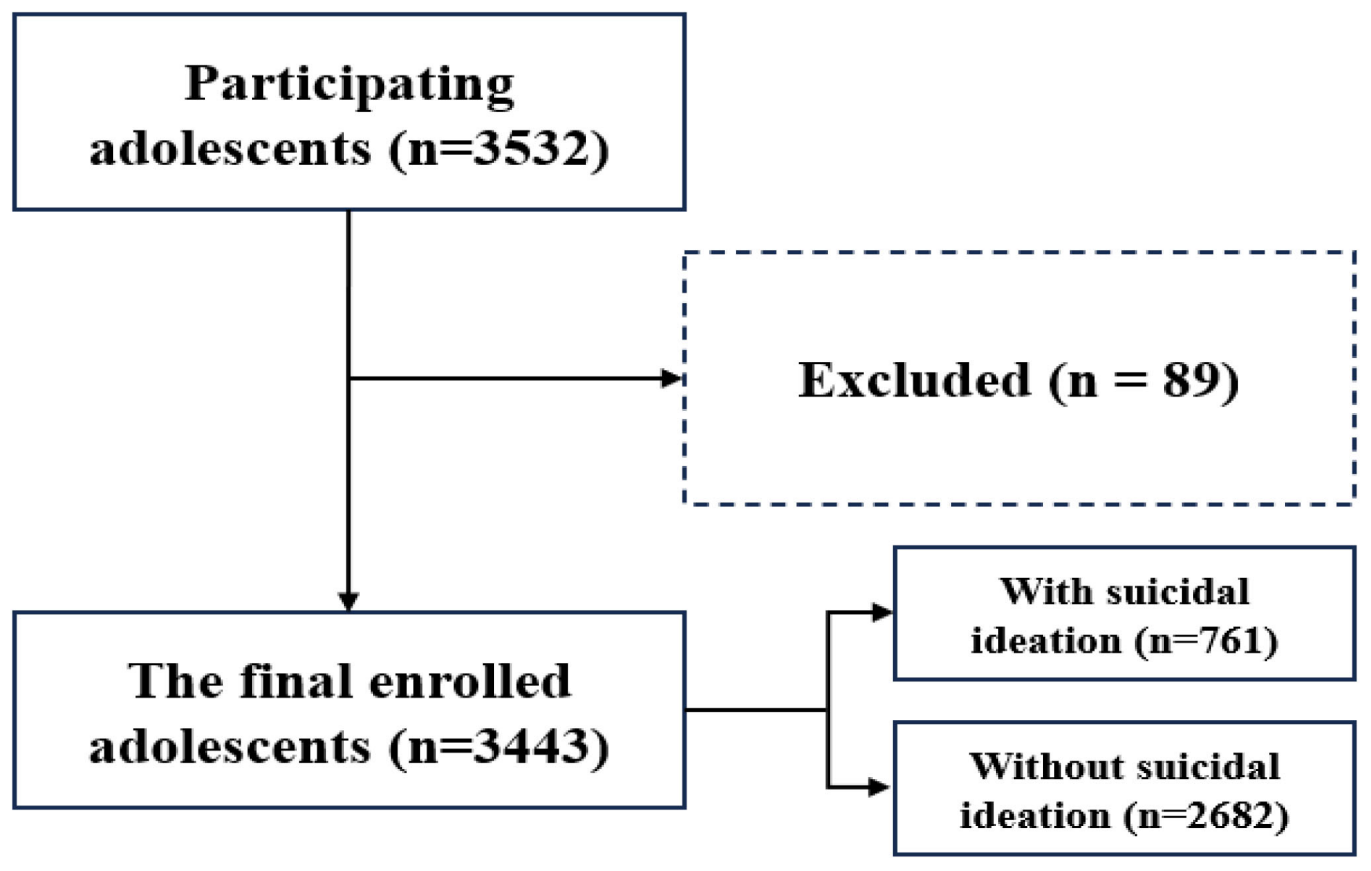
Flow chart of this study.

A cross-sectional survey was conducted between May and June 2023 to assess the mental health status of urban adolescents in Lianyungang, China. To ensure the representativeness of the sample, we assigned unique identification numbers to the secondary schools in the urban area of Lianyungang. We then used a lottery method to randomly select five schools. Subsequently, unbiased school teachers, who were not affiliated with the research team, carried out a random selection of students from these five schools to ensure the independence, blindness, and randomness of participant selection. General demographic and scale information about the participants was collected through the online questionnaire platform “wenjuanxing” (https://www.wjx.cn/app/survey.aspx). The survey was carried out with the consent of the respective school authorities, parents, and students. The participant recruitment process is illustrated in **Figure 1**. In the present study, we also collected the patient health questionnaire-9 (PHQ-9) and the generalized anxiety disorder-7 (GAD-7) scores from adolescent mothers.

### Ethical considerations

Prior to the survey, students were provided with detailed information about the study’s objectives, procedures, data confidentiality, and anonymity. Participation in the survey was completely voluntary, and students had the freedom to discontinue completing the questionnaire at any time if they so desired. The project was conducted with the approval of the Ethics Committee of the Fourth People’s Hospital in Lianyungang. Given the sensitive nature of the research topic, additional protective measures were implemented. All research staff received specialized training to address potential emotional distress. We established a confidential referral system to provide professional mental health support for students exhibiting high-risk behaviors. Anonymous coding was used during data collection to ensure personal information was stored separately from survey data. Furthermore, informed consent was obtained from all participants or their guardians. For minor participants, consent was obtained from both the student and their guardian.

### Data collection

The research team developed a semi-structured questionnaire to collect sociodemographic information from participants. The questionnaire covered aspects such as age, gender, presence of single-parent households (yes/no), quality of parental relationships (harmonious/moderate/troubled), and annual household income (high/good/fair/poor). To assess the parental relationship, we asked: ‘How would you describe the relationship between your father and mother?’ The options were: harmonious (parents generally have a good relationship with few quarrels), moderate (parents have a generally good relationship with occasional quarrels), and troubled (parents have a poor relationship with frequent quarrels). The income brackets were defined as follows: “poor” for annual household incomes below CNY 50,000, “fair” for incomes between CNY 50,000 and CNY 100,000, “good” for incomes between CNY 100,000 and CNY 200,000, and “high” for incomes of CNY 200,000 or more.

### Suicidal ideation

Participants were evaluated for the presence of suicidal ideation using the PHQ-9. The PHQ-9 is a 4-point scale ranging from 0 to 3, with 0 representing “not at all”, 1 representing “several days”, 2 representing “more than half the days”, and 3 representing “nearly every day” (27). Psychological status was evaluated over a two-week period; the ninth item “Thoughts that you would be better off dead or of hurting yourself in some way” reflected the absence of suicidal ideation with a score of 0 and the presence of such thoughts with a score of ≥ 1 (28–30). The PHQ-9 scale has demonstrated good reliability and validity in Chinese and adolescent populations (31–33).

### Anxiety symptoms

The GAD-7 instrument is a commonly used self-report scale for measuring individual anxiety symptoms over a two-week period. It utilizes a 4-point scale (ranging from 0 to 3) to assess the severity of symptoms, with 0 indicating “not at all”, 1 indicating “several days”, 2 indicating “more than half the days”, and 3 indicating “almost every day”. This scale has a total score range of 0–27, providing insights into the level of anxiety experienced by an individual (34, 35). A study by Spitzer et al. found that the sensitivity and specificity of the GAD-7 scale with a cut-off of 10 points were 89.0% and 82.0%, respectively, which was an appropriate criterion for detecting anxiety symptoms; therefore, we adopted this criterion and considered no anxiety symptoms when the total score was < 10 and anxiety symptoms when the total score was ≥ 10 (36). The GAD-7 scale has been well-validated for reliability and validity in Chinese populations, including adolescents (37–39).

### Social support

Perceived social support was assessed using the perceived social support scale (PSSS), which is composed of 12 items that are subdivided into three subscales of family, friends, and significant others on a 7-point scale of 1–7, from “very strongly disagree” to “very strongly agree”, with higher scores indicating higher perceived support (40). As measured by Zimet et al., the Cronbach’s alpha coefficients for the family, friends, and significant others subscales were determined to be 0.87, 0.85, and 0.91, respectively (40). The PSSS has been widely applied in research on Chinese populations (23, 41, 42).

### Statistical analysis

**Figure 2:**
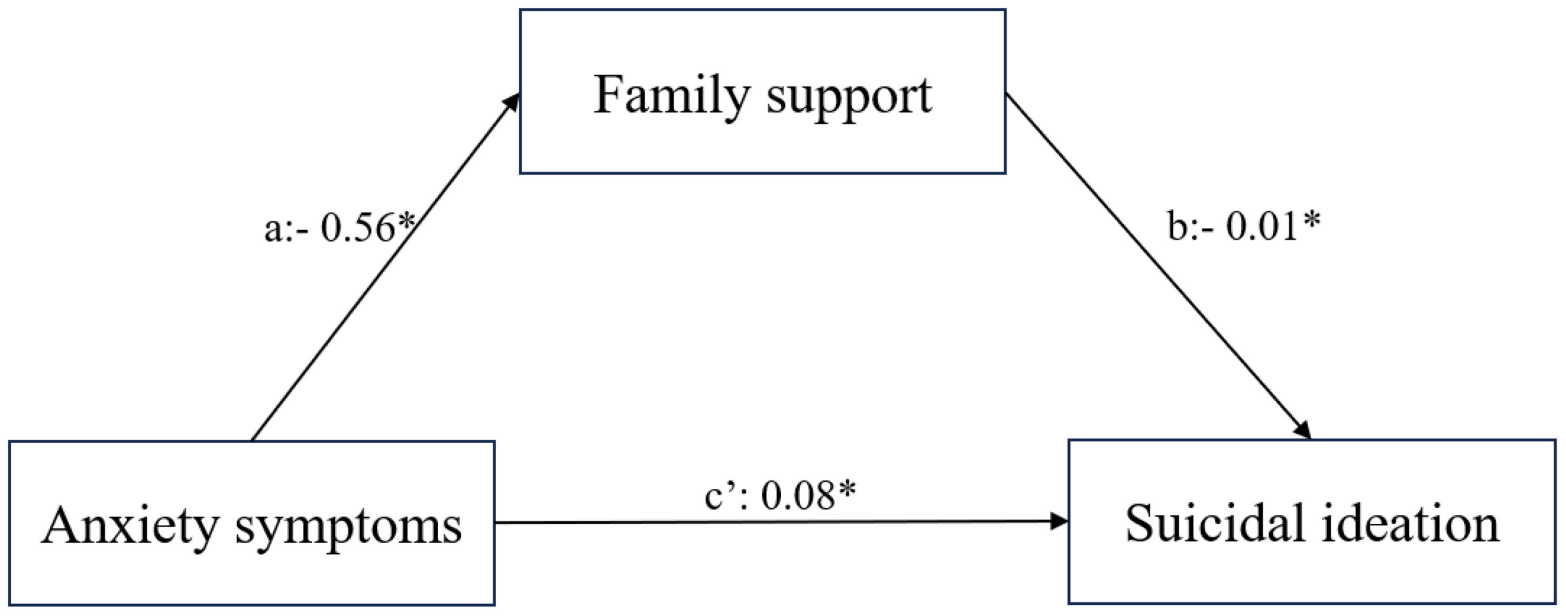
The model showing family support mediating the relationship between anxiety symptoms and suicidal ideation in adolescents. *P < 0.05.

Statistical Package for the Social Sciences (SPSS) software (version 23.0) was used for statistical analysis. Age and other continuous variables were evaluated using Student’s t-test or independent samples t-test. Mean and standard deviation (SD) were used to assess central tendency and dispersion. Categorical variables were compared via descriptive statistics and the chi-square test. Spearman’s correlation coefficient was applied to assess the correlation between the variables. Logistic regression was used to identify risk factors for suicidal ideation. Multiple regression analysis was conducted to examine the correlation between anxiety symptoms and social support, while controlling for potential confounding factors. The mediating role of anxiety symptoms in the link between social support and suicidal ideation was examined using model 4 of PROCESS v.3.5. To ensure sufficient statistical power, we used G*Power 3.1 for sample size estimation. Based on the anticipated effect size (0.8), significance level (α=0.05), and desired power (1-β, 0.8). we selected a conservative estimate of 0.9 for the ratio var1/var0. The significance level was set at P < 0.05. **Figure 1** in the manuscript was created using the Processon online diagram tool (https://www.processon.com/diagrams), and **Figure 2** was created using JAMOVI 2.3, along with R version 4.3.0.

## Results

### Characteristics of study participants

Of 3532 questionnaires collected, 3443 were found to be valid after excluding 89 for incomplete entries (shown in **Figure 1**). The surveyed adolescents, aged 12–18, had an average age of 14.88 years (SD 1.59), and an average PSSS score of 65.0 (15.18). Suicidal ideation was present in 22.1% (n = 761) and anxiety symptoms in 10.2% (n = 351). Parental relationships were self-reported as harmonious (72.0%), moderate (24.2%), or troubled (3.8%). Single-parent households made up 10.0%; annual household incomes were categorized as high (25.4%), good (20.1%), fair (51.9%), and poor (2.6%).

### Comparison of demographic characteristics and parameters between individuals with suicidal ideation and those without suicidal ideation

As shown in **Table 1**, a significantly higher proportion of female adolescents reported suicidal ideation compared with males (27.9% VS 16.9%, P < 0.001). The proportion of all individuals with accompanying anxiety symptoms was significantly higher than those without anxiety symptoms (77.2% VS 15.8%, P < 0.001). Moreover, among adolescents with suicidal ideations, there was an increased percentage of depressive symptoms in their mothers (32.9% VS 21.6%, P = 0.002), with no significant difference in anxiety symptoms (23.9% VS 22.0%, P = 0.654).

**Table 1:** Social demographics and clinical characteristics of individuals with suicidal ideation and without suicidal ideation.

Variables	With (n = 761)	Without (n = 2682)	χ^2^/t	P
Age (years), mean (SD)	14.68 (1.54)	14.94 (1.60)	4.198^a^	<0.001
Sex, n (%)			60.870^b^	< 0.001
Male	307 (16.9)	1511 (83.1)		
Female	454 (27.9)	1171 (72.1)		
GAD, n (%)			689.318^b^	< 0.001
Yes	271 (77.2)	80 (22.8)		
No	490 (15.8)	2602 (84.2)		
PHQ-9 of mothers, n (%)			9.803^b^	0.002
Yes	46 (32.9)	94 (67.1)		
No	715 (21.6)	2588 (78.4)		
GAD-7 of mothers, n (%)			0.200^b^	0.654
Yes	26 (23.9)	83 (76.1)		
No	735 (22.0)	2599 (78.0)		
PSSS, mean (SD)				
Family	17.15 (5.57)	23.07 (4.90)	26.540^a^	< 0.001
Friends	18.46 (5.53)	22.49 (4.87)	18.183^a^	< 0.001
Significant other	17.76 (5.31)	22.74 (4.80)	23.325^a^	< 0.001
Single-parent households, n (%)			18.546^b^	< 0.001
Yes	108 (31.2)	238 (68.8)		
No	653 (21.1)	2444 (78.9)		
Parental relationship, n (%)			276.951^b^	< 0.001
Harmonious	369 (14.9)	2110 (85.1)		
Moderate	325 (39.1)	507 (60.9)		
Troubled	67 (50.8)	65 (49.2)		
Annual household incomes, n (%)			57.458^b^	< 0.001
Better	144 (16.4)	732 (83.6)		
Good	126 (18.2)	566 (81.8)		
Fair	452 (25.3)	1334 (74.7)		
Poor	39 (43.8)	50 (56.2)		

SD, standard deviations; GAD, the Generalized Anxiety Disorder-7; PSSS, Perceived Social Support Scale. ^a^independent samples t-test; ^b^chi-square test.

Compared with adolescents without suicidal ideation, those with suicidal ideation had lower scores on the family, friends, and significant other subscales of the PSSS (all P < 0.05). Adolescents in single-parent families exhibited a higher prevalence of suicidal ideation compared with those in non-single-parent families (31.2% VS 21.1%, P < 0.001). Similarly, a higher prevalence of suicidal ideation was observed among adolescents reporting troubled parental relationships and with poor annual family economic income, as compared to those in harmonious parental relationships and with high annual family income (50.8% VS 14.9%; 43.8% VS 16.4%, respectively, all P < 0.001).

### Analysis of risk factors associated with suicidal ideation in adolescents

Among the 3,443 adolescents, the proportions reporting of suicidal ideation on a scale of 0-3 were 2682 (77.9%), 609 (17.7%), 84 (2.4%), and 68 (2.0%), respectively.

Binary logistic regression analyses indicated that female sex (OR = 1.788, 95% CI: 1.467–2.177, P < 0.001), anxiety symptoms (OR = 10.035, 95% CI: 7.441–13.534, P < 0.001), total PHQ-9 scores of mothers (OR = 1.040, 95%CI: 1.003 – 1.078, P = 0.034), total GAD-7 scores of mothers (OR = 0.958, 95%CI: 0.919 – 0.998, P = 0.039), and moderate parental relationships (OR = 2.042, 95% CI: 1.630–2.557, P < 0.001) were risk factors for suicidal ideation, while family support was protective (OR = 0.888, 95% CI: 0.859–0.918, P < 0.001). However, the results indicated that age, support from friends or significant other, single-parent families, and annual household income were not risk factors for adolescent suicidal ideation (all P > 0.05) (**Table 2**).

**Table 2:** Risk factors related to the prevalence of suicidal ideation.

Variables	B	OR	95%CI	P
Age	- 0.042	0.958	0.900 – 1.021	0.187
Females	0.581	1.788	1.467 – 2.177	< 0.001
GAD	2.306	10.035	7.441 – 13.534	< 0.001
Total PHQ-9 scores of mothers	0.039	1.040	1.003 – 1.078	0.034
Total GAD-7 scores of mothers	- 0.043	0.958	0.919 – 0.998	0.039
PSSS				
Family	- 0.119	0.888	0.859 – 0.918	< 0.001
Friends	0.007	1.007	0.976 – 1.040	0.652
Significant other	- 0.036	0.965	0.925 – 1.006	0.093
Single-parent households	0.086	1.090	0.789 – 1.507	0.601
Parental relationship				
Harmonious		1		
Moderate	0.714	2.042	1.630 – 2.557	< 0.001
Troubled	0.240	1.271	0.762 – 2.120	0.358
Annual household income				
Better		1		
Good	- 0.041	0.960	0.703 – 1.311	0.799
Fair	0.031	1.032	0.802 – 1.327	0.806
Poor	0.113	1.119	0.618 – 2.027	0.710

GAD, the Generalized Anxiety Disorder-7; PSSS, Perceived Social Support Scale.

### The mediating role of family support between anxiety symptoms and suicidal ideation

Spearman correlation analysis revealed a positive correlation between suicidal ideation and anxiety symptoms (r = 0.603, P<0.001), as well as a negative correlation with family support (r = –0.426, P<0.001). Further, suicidal ideation was considered as the dependent variable, and multiple regression analysis was utilized to examine the total effects (β=0.585, 95%CI:0.049−0.054, R^2^ = 0.406) and direct effects (β=0.525, 95%CI:0.044−0.049, R^2^ = 0.424) of total anxiety symptom scores on suicidal ideation. Family support was used as the dependent variable, and multiple regression analysis was conducted to analyze the effect of total anxiety symptom scores on family support (β=–0.366, 95%CI: –0.474 to –0.404, R^2^ = 0.321). The path diagram of the regression model is shown in **Figure 2**.

Furthermore, as shown in **Table 3**, family support showed a partial mediation effect in the relationship between anxiety symptoms and suicidal ideation, with the mediating effect contributing 9.28% to the total effect.

**Table 3:** Indirect and total effects for the mediation analyses.

Effect	95%CI				
	Estimate	Lower	Upper	β	Mediation
GAD-7 total score → Family support → Suicidal ideation (indirect)	0.008*	0.006	0.010	0.063	9.28%
GAD-7 total score → Family support	-0.560*	-0.601	-0.517	-0.467	–
Family support → Suicidal ideation	-0.015*	-0.018	-0.011	-0.134	–
GAD-7 total score → Suicidal ideation (direct)	0.080*	0.073	0.086	0.612	90.72%
GAD-7 total score → Suicidal ideation (Total)	0.088*	0.082	0.093	0.674	100%

GAD, Generalized Anxiety Disorder-7; * P < 0.001.

## Discussion

This study reveals a 22.1% prevalence of suicidal ideation among Chinese adolescents. Female sex, anxiety symptoms, the mothers exhibited depressive symptoms, insufficient family support, and parental relationships heightened the risk. Moreover, family support mediates the relationship between anxiety symptoms and suicidal ideation.

The prevalence of suicidal ideation among Chinese adolescents was found to be 22.1% in our study. It is important to note that the research on the prevalence of suicidal ideation among adolescents has yielded inconsistent findings across different cultural backgrounds, countries, and periods. For instance, according to Peng et al., the prevalence of suicidal ideation among Chinese adolescents was 23.5% (43). The prevalence of self-reported suicidal ideation in Polish adolescents was 24.66% (9), and in Tunisian adolescents was 26.9% (16). A study examining the prevalence of suicidal ideation among U.S. adolescents from 1991 to 2019 found that it ranged from 19.4% to 15.8% (44). A meta-analysis examining data from 1989 to 2018 found that the 12-month prevalence of suicidal ideation among adolescents was estimated to be 18% (45). During COVID-19, the prevalence ranged from 29.7% to 31.3% (46). Although these results vary, these alarming statistics highlight the urgent need for effective strategies to prevent and address suicidal ideation in this vulnerable group.

One important finding of our study is the association of female sex with suicidal ideation. Specifically, females were found to have a higher risk of experiencing more suicidal ideation. This result aligns with previous research indicating that females are more vulnerable to suicidal ideation (47–49). Previous studies have elucidated the ways in which females are more prone to suicidal ideation. Ho et al. found pubertal hormonal changes and social stressors to have an impact on neurobiologically sensitive female adolescents (50). Giletta et al. identified the hypothalamic–pituitary–adrenal axis stress response to be a risk factor in females (51). These insights clarify the link between female sex and suicidal ideation in neurobiological and psychosocial contexts. Nevertheless, understanding the risk factors associated with suicidal ideation in females remains complex, underscoring the need for continued research to explore the underlying factors contributing to this gender difference.

Anxiety symptoms significantly correlate with suicidal ideation in adolescents, as evidenced by existing studies (15, 52, 53). Research, including one study with 7054 adolescents averaging 15.8 years, highlights the link between generalized anxiety symptoms and suicidal thoughts (54). Similarly, a cross-sectional study among high schoolers revealed this moderate association (55). Moreover, a systematic review and meta-analysis demonstrated a significant association between higher levels of social anxiety and more frequent occurrences of suicidal ideation (52). Multiple multilevel analyzed studies have consistently highlighted a significant correlation between learning anxiety and suicidal ideation (56). Furthermore, studies investigating the relationship between anxiety symptoms and suicidal ideation consistently emphasize the importance of addressing anxiety symptoms as a crucial preventive measure against suicidal ideation in adolescents.

Our study highlights the significant link between adolescents’ suicidal ideations and their mothers’ depressive symptoms. Consistent with prior research, maternal depression emerges as a potential risk factor for adolescent suicidality (57, 58). This complex relationship warrants a comprehensive approach that considers other influencing factors and the reciprocal effects of maternal depression and adolescent suicidality (59, 60). Consequently, our findings and supporting literature emphasize the importance of early identification and family-centered interventions to mitigate the risk of suicidal ideations in adolescents.

In addition, lacking family support and parental relationships were found to be associated with an increased risk of suicidal ideation. These findings are in line with previous research highlighting the importance of family relationships and support as protective factors against suicidal ideation (21, 61), while family conflict exacerbates the severity of suicidal ideation (62). These studies also underscore the crucial role of family dynamics and support systems in promoting adolescent mental health (63, 64).

Furthermore, the present study found that family support had a partial mediating effect on the relationship between anxiety symptoms and suicidal ideation, suggesting that strengthening family support systems may serve as a protective factor, which is consistent with previous study (21, 65). For example, a previous longitudinal study demonstrated that high levels of parental support significantly reduced the development of anxiety symptoms in adolescents, thereby indirectly decreasing the emergence of suicidal ideation (66). Another study found that family support played a protective role in mitigating anxiety symptoms and reducing suicidal ideation (67). These findings suggest that enhancing family support can effectively alleviate psychological stress and negative emotions in adolescents, thereby reducing the occurrence of suicidal ideation. However, it is important to note that family support alone is not sufficient to explain fully the relationship between anxiety symptoms and suicidal ideation. Other factors, such as individual coping mechanisms, peer relationships, and access to mental health resources, may also contribute to the complex interplay between anxiety and suicidal ideation in adolescents, and also require attention.

In recent years, China has made significant progress in adolescent mental health policies (68), as evidenced by the 2012 Mental Health Law and the 2019 guidelines on strengthening mental health education in schools. However, challenges remain in implementing these policies and allocating resources, particularly in rural and underdeveloped areas. Our research findings suggest the formulation and implementation of evidence-based policies and interventions. To address the higher risk of suicidal ideation among female adolescents, it is crucial to enhance tailored mental health support. Specifically, schools can implement gender-specific mental health education programs, provide safe spaces for discussion, and train teachers to identify high-risk female students. Moreover, improving accessibility to treatment for anxiety symptoms is essential to reduce the likelihood of developing suicidal ideation. Healthcare providers should consider establishing on-campus counseling stations to offer low-threshold mental health services and conduct anxiety management workshops. Strengthening family support programs can play a vital role in mitigating the risk by providing guidance and support, particularly for those with troubled parental relationships. This can be achieved through organizing parental education courses, family counseling services, and parent-child activities. Policymakers should consider incorporating mental health education into school curricula and increasing funding for school-based mental health services. By implementing these measures, we can work towards creating a safer and more supportive environment for adolescent mental health.

Although our study provides valuable insights into the prevalence and risk factors of suicidal ideation among Chinese adolescents, it is important to acknowledge some limitations. The data collected in this study relied on self-report measures, which may be subject to recall bias. To address this limitation in future research, studies could employ multi-informant data collection methods, incorporating reports from parents and teachers, as well as medical records, to enhance the accuracy and reliability of the data. Additionally, the study design was cross-sectional, limiting our ability to establish causal relationships. Future longitudinal studies are needed to explore further the complex interactions between various risk factors and suicidal ideation among adolescents. Specifically, future research should consider conducting longitudinal studies with regular assessments, such as every six months or annually, over a period of 3-5 years. This longitudinal approach would facilitate the establishment of causal relationships between risk factors and suicidal ideation, as well as observe how these relationships evolve over time. Thirdly, due to constraints in the study design, we were unable to include some important factors that may influence suicidal ideation, such as rural areas, circadian cycle regulation, drug use, depression, stress, and hormonal regulation. These factors could potentially have significant impacts on suicidal ideation among adolescents. Future studies should consider incorporating these variables to gain a more comprehensive and in-depth understanding of the risk factors for suicidal ideation in adolescents, thereby informing more targeted intervention strategies.

## Conclusions

In conclusion, this study highlights the high prevalence of suicidal ideation among adolescents and identifies several risk factors associated with its occurrence. A partial mediating role of family support was found in the relationship between anxiety symptoms and suicidal ideation. Suicidal ideation among adolescents requires attention, and families, schools and policy makers should collaborate to plan measures to prevent suicidal ideation among adolescents. Our findings provide a foundation for the formulation and implementation of evidence-based policies and interventions, including tailored mental health support for female adolescents, improved accessibility to treatment for anxiety symptoms, and strengthened family support programs. By implementing these multifaceted strategies, we can work towards creating a safer and more supportive environment for adolescent mental health, effectively reducing the risk of suicidal ideation.

## Data availability statement

The original contributions presented in the study are included in the article. Further inquiries can be directed to the corresponding authors.

## Ethics statement

The project was conducted with the approval of the Ethics Committee of the Fourth People’s Hospital in Lianyungang. The studies were conducted in accordance with the local legislation and institutional requirements. Written informed consent for participation in this study was provided by the participants’ legal guardians/next of kin.

## Author contributions

XB: Writing – original draft, Data curation, Methodology, Software. TG: Writing – original draft, Data curation, Methodology, Software. LX: Data curation, Formal Analysis, Investigation, Writing – original draft. WC: Data curation, Investigation, Methodology, Writing – original draft. LL: Data curation, Investigation, Writing – original draft. HY: Conceptualization, Funding acquisition, Investigation, Writing – review & editing, Formal Analysis, Supervision. XZ: Conceptualization, Funding acquisition, Supervision, Writing – review & editing, Methodology, Validation.
